# Psychological Treatment for Pediatric Feeding Disorder (PFD) and Avoidant/Restrictive Food Intake Disorder (ARFID)

**DOI:** 10.1002/eat.70101

**Published:** 2026-05-24

**Authors:** Colleen T. Lukens, Robert M. Dempster, Kamryn T. Eddy, Hayley Estrem, Jessie E. Menzel, Richard J. Noel, Jaclyn L. Pederson, Ana L. Ramirez, Cuyler Romeo, William G. Sharp, Jennifer J. Thomas, Nancy L. Zucker, Hana F. Zickgraf

**Affiliations:** ^1^ Children's Hospital of Philadelphia Philadelphia Pennsylvania USA; ^2^ Nationwide Children's Hospital Columbus Ohio USA; ^3^ Massachusetts General Hospital Boston Massachusetts USA; ^4^ University of North Carolina Wilmington North Carolina USA; ^5^ Equip California USA; ^6^ Duke Children's Hospital and Health Center Durham USA; ^7^ Feeding Matters Phoenix Arizona USA; ^8^ UC San Diego Health San Diego California USA; ^9^ Banner‐University Medical Center South Tucson Arizona USA; ^10^ Emory Medical School, Children's Healthcare of Atlanta Atlanta Georgia USA; ^11^ Duke University Health Center Durham North Carolina USA; ^12^ Rogers Behavioral Health Oconomowoc Wisconsin USA

## Abstract

**Objective:**

To produce a consensus statement on the psychological treatment of feeding/eating aversions seen in pediatric feeding disorder (PFD) and avoidant/restrictive food intake disorder (ARFID), diagnoses that share common symptoms and psychological mechanisms but have historically been addressed separately in the literature.

**Method:**

To help bridge the gaps between ARFID and PFD treatment and research, we convened a group of experts to clarify and describe the psychological interventions for treating learned food avoidance. The current manuscript summarizes the conclusions of the meeting.

**Result:**

The primary outcome of the consensus process was a description of a common core approach to psychological intervention across the two fields: Exposure to food and the feeding/eating experience with the goal of increasing the volume and variety of food and fluid consumed. Although our consensus process identified some differences in the mode of delivery and incorporation of specific treatment elements, the consensus panel agreed that these differences are related to three primary factors: the severity of clinical impairment, age and developmental status of the patient, and the specific feeding and eating behaviors targeted, rather than to the ARFID/PFD diagnostic distinction.

**Discussion:**

Building on the consensus statement, we discuss other considerations in preparing for and delivering exposure‐based treatment, including multidisciplinary assessment, readiness for treatment, and systemic factors that influence access and engagement.

## 1 Introduction

Diagnostic criteria for both pediatric feeding disorder (PFD) and avoidant/restrictive food intake disorder (ARFID) involve difficulties with being fed or eating an age‐ and developmentally‐ appropriate volume and/or variety of food, resulting in medical, nutritional, and/or psychosocial impairment (Estrem et al. 2025; Goday et al. 2019; Eddy et al. 2019). The outward behavioral manifestation of PFD and ARFID is similar, in that individuals with these conditions engage in avoidance of food and feeding/eating situations which results in impairments in weight/growth, nutrition, and/or psychosocial functioning. The ARFID and PFD diagnoses were developed in different clinical contexts and only recently has there been an effort to refine the criteria for these disorders to facilitate differential and comorbid diagnosis (e.g., Estrem et al. 2025).

There is emerging agreement that the PFD diagnosis is best applied to children whose current feeding/eating difficulties are maintained by medical or feeding skill‐related factors, whereas ARFID describes food avoidance behavior that is maintained by aversive emotional responses to food and being fed or eating. However, this distinction is not always clear. For example, many patients with PFD also meet criteria for ARFID because their food avoidance is influenced by psychological aversions as well as medical and feeding skill factors. In addition, many patients convert from a PFD to an ARFID diagnosis when the medical or skill conditions are appropriately managed but emotional aversions remain (Estrem et al. 2025).

The feeding/eating difficulties associated with ARFID can be characterized by three presentations: (1) aversion to the sensory properties of food, (2) poor appetite or limited interest in food, and (3) fear of one or more aversive consequences of eating, such as choking, vomiting, becoming ill, or having an allergic reaction (APA 2022). In older patients who communicate verbally, it is usually possible to identify these primary presentations; however, in children who are still in the developmental stage where feeding/eating skills, behaviors, and emotional responses are becoming established, the feeding/eating presentation may not be as well differentiated. In young children or children with communication delays or deficits, the topography of mealtime behaviors might be indicative of these subtypes. For example, gagging or food selectivity could reflect an aversion to the sensory properties of food, consuming a limited volume of food might suggest poor appetite or limited interest in food, and food avoidance could indicate a fear of aversive consequences of feeding/eating.

ARFID and PFD are both common problems warranting intervention. The general population prevalence for ARFID is estimated to be as high as 4.5% (Nicholls‐Clow et al. 2024), and 22.5% of patients presenting for eating disorder‐focused care before the diagnosis was published in 2013 met retroactively applied criteria for ARFID (Nicely et al. 2014). In a retrospective cohort study examining the occurrence of PFD between 2009 and 2014, authors estimated an annual prevalence as high as 1 in 23 children under 5 years of age (Kovacic et al. 2021).

The current literature on treatment outcome is limited and based on case studies, case series, retrospective reports from naturalistic clinical settings, single‐arm clinical trials, and two randomized feasibility trials, given the relative novelty of the PFD and ARFID diagnoses (Breiner et al. 2024a; Brigham et al. 2018; Dalle Grave and Sapuppo 2020; Hellner et al. 2025; Kambanis and Thomas 2023; Lock, Robinson et al. 2019; Lock, Sadeh‐Sharvit, and L'Insalata 2019; Lukens and Silverman 2014; Medina‐Tepal et al. 2023; Palmer et al. 2025; Sharp et al. 2010, 2017). This literature treats ARFID and PFD as separate diagnoses, with interventions described as targeting either ARFID or PFD (Table 1). ARFID treatment as described in the available literature utilizes general principles of cognitive behavioral therapy, acceptance and commitment therapy, family‐based treatment, and parent training that are established as evidence‐based for eating and anxiety disorders and behavioral problems in children (e.g., Howard et al. 2023; Shimshoni and Lebowitz 2020; Willmott et al. 2024). Psychosocial interventions for PFD as currently examined in the literature incorporate tenets of behavioral therapy and parent management therapies, both well‐established treatment philosophies for addressing behavioral difficulties commonly seen in early childhood (e.g., sleep difficulties, toileting difficulties, uncooperative behavior; Lukens and Silverman 2014; Sharp et al. 2017).

**TABLE 1:** Summary of published treatment outcome data (pilot studies, naturalistic studies, and randomized controlled trials) for treatments aimed at psychological feeding/eating aversions in populations diagnosed with ARFID and/or PFD.

Treatment program	Aim, approach, theoretical orientation	Intended population/frequency of treatment	Evidence‐base
**Programs evaluated in populations diagnosed with ARFID or in ARFID‐focused treatment settings**			
Cognitive behavioral therapy for ARFID (CBT‐AR)	*Aim*: Focuses on exploring the sensory properties of new foods, tolerating interoceptive sensations of fullness, and challenging eating‐related fears and reducing avoidance. Prioritizes weight gain/nutritional stabilization early in treatment. *Approach*: Incorporates interventions such as regular eating, introducing a supplement, and increasing portion sizes. Uses repeated, presentation‐specific exposure. Including caregivers in treatment is recommended for children and adolescents/young adults with low weight or significant weight loss *Theoretical orientation*: Cognitive behavioral	Youth ages 8–17 and adults Weekly outpatient Child‐directed intervention with caregiver support	Burton Murray et al. (2022), Burton‐Murray et al. (2024), Palmer et al. (2025), and Thomas and Eddy (2018).
Inhibitory learning‐focused exposure	*Aim*: designed to target patients' expectancies about the safety and/or tolerability of engaging with avoided foods/eating situations *Approach*: Utilizes presentation‐specific exposure. Therapeutic meals and experiential and therapeutic groups. Family involvement in the form of regular family sessions during intensive treatment is aimed at supporting parents to carry out exposures and follow meal plan/regular eating on evenings and weekends *Theoretical orientation*: Cognitive behavioral	Youth ages 7–17 Intensive treatment programs (partial hospitalization) Child‐directed intervention with caregiver support	Dumont et al. (2019) and Lane‐Loney et al. (2022).
Family‐based treatment for ARFID (FBT)	*Aim*: Centered on supporting caregivers in feeding their child age‐ and developmentally appropriate diets by reducing accommodation, using behavioral strategies, and externalizing the illness. *Approach*: Family‐based therapy with the addition of child‐directed exposure, cognitive techniques, and age‐downward extension of behavioral techniques *Theoretical orientation*: Adapted for ARFID from family‐based treatment for adolescent anorexia nervosa	Ages 5–12 Caregiver‐directed	Eckhardt et al. (2019), Hellner et al. 2025, Lien et al. (2025), Lock (2021), Lock, Robinson et al. (2019); Lock, Sadeh‐Sharvit, and L'Insalata (2019), and Rosania and Lock (2020)
Feelings and body investigators—ARFID division (FBI‐ARFID)	*Aim*: To teach children to differentiate, understand, and adaptively respond to bodily sensations, across ARFID presentations *Approach*: Uses psychoeducation (in the form of cartoons), interoceptive exposure, and acceptance techniques; based on the theory that ARFID arises from fear and avoidance of interoceptive sensations *Theoretical orientation*: Cognitive behavioral and acceptance based	Children ages 4–10 Children and caregivers participate together, 11–15 sessions	Zucker et al. (2019)
ARFID parent training protocol (ARFID‐PTP)	*Aim*: Teach parents to deliver evidence‐based behavioral interventions *Approach*: Behavioral interventions include establishing regular eating (mealtime hygiene/appetite optimization), using behavioral strategies (i.e., rewards, attention) to manage contingencies, and delivering exposure. Presentation‐specific modules provide psychoeducation and adapted interventions for each presentation *Theoretical orientation*: Parent‐therapist behavioral model	Parents of children ages 5–12 Individual teletherapy format; caregiver directed Two 2 h sessions	Breiner et al. (2024a) and Breiner et al. (2024b).
Supportive parenting for anxious childhood emotions (SPACE‐ARFID)	*Aim*: Promote the child's food‐related flexibility and systematically reduce accommodation in specific areas while increasing supportive responses and managing parents' food‐related stress *Approach*: Focuses on parental responses to problematic feeding/eating behaviors with no child‐directed interventions; the target for change is parents' responses to their child's behavior *Theoretical orientation*: Parent‐therapist behavioral model	Parents of children ages 6–14 12 weekly 60‐min sessions Caregiver directed	Shimshoni and Lebowitz (2020) and Shimshoni et al. (2020).
Picky Eaters Clinic	*Aim*: Improve selective eating *Approach*: Incorporates psychoeducation on mealtime hygiene and appetite optimization, overview of behavioral principles, group support, planning exposures and rewards, role plays and problem solving, and relapse prevention *Theoretical Orientation*: Parent‐therapist behavioral model	Parents of children ages 4–11 Group based, seven 90‐min sessions Caregiver directed	Dahlsgaard and Bodie (2019)
**Programs evaluated in populations diagnosed with PFD or in PFD‐focused treatment settings**			
Parent training—feeding (PT‐F)	*Aim*: Addresses feeding problems and associated parent stress. Equips families with behavioral strategies to address feeding problems. *Approach*: Incorporates parent education, function of problematic feeding behavior, ABC model, prevention strategies, and reinforcement strategies. *Theoretical orientation*: Behavioral	Parents of children with ASD ages 2–7 years Individual outpatient therapy, caregiver‐directed, 9 sessions over 16 weeks	Johnson et al. (2015)
Integrated eating aversion treatment (iEat)	*Aim*: Increase food acceptance and food volume *Approach*: Behavioral intervention involving escape extinction, reinforcement procedures, and formalized meal structure Theoretical *orientation*: Behavioral	Children ages 1–6 years Individual outpatient therapy, child‐directed, 5 days	Sharp et al. (2016)
Behavioral/behavior analytic feeding therapy	*Aim*: Increase food acceptance and food volume to stabilize nutrition, weight, and growth and address food selectivity, chewing, packing, food refusal, and tube weaning *Approach*: Behavioral intervention involving escape extinction, reinforcement procedures, and formalized meal structure. Multidisciplinary approach aims to address skill deficits, medical problems, and nutritional impact as well as avoidant behavior/learned aversions. *Theoretical orientation*: Behavioral	Parents of children ages 1–6 years or older children with intellectual and developmental delays Individual outpatient therapy; caregiver directed Inpatient or partial hospitalization, 6–10 weeks; caregiver directed	Sharp et al. (2017) and Williams and Seiverling (2023).

As illustrated in Table 1, treatment research has historically focused on populations identified as having either ARFID or PFD, despite the target of the intervention in both cases being aversions to feeding/eating resulting in inadequate nutritional or caloric intake and/or family and psychosocial impairment. Given the high degree of comorbidity and diagnostic cross‐over between ARFID and PFD (Estrem et al. 2025), the siloed treatment approaches may lead to confusion for families and referring providers (Sharp et al. 2017). To help bridge the gaps between ARFID and PFD treatment and research, we convened a group of experts to clarify and describe the psychological interventions for treating learned food avoidance. This article summarizes the results of that consensus meeting, highlighting the similarities in the psychological treatments for PFD and ARFID. The authors present a summary statement, integrating currently available evidence and expert consensus.

## 2 Method

In response to the ongoing need for collaboration between the PFD and ARFID fields, the Feeding Matters Research Initiative Task Force hosted the 2024 PFD‐ARFID Psychology Summit comprised of a three‐hour live virtual education webinar on PFD and ARFID psychological interventions followed by a two‐day facilitated consensus meeting of clinical psychologists specializing in the research on and treatment of PFD and/or ARFID and meeting at least 4 of the criteria below:

- Consensus Experience—served as an author on at least one of the following PFD/ARFID consensus publications—(Eddy et al. 2019; Estrem et al. 2025; Goday et al. 2019).
- Research Expertise—publication of PFD and/or ARFID related articles in a peer reviewed journal within the 3 years prior to the convening
- Leadership Experience—fulfilling appointed or elected positions in national organizations dedicated to care advancement in PFD and/or ARFID or related field
- Clinical Expertise—currently providing at least 20 h of direct patient care per month or 20 h of clinical activities which may include any of the following: supervision, group fabrication, clinical teaching, clinical consultation, and program development/maintenance
- Professional Endorsement—nominated by a 2023 PFD‐ARFID consensus member to represent the field

Nine clinical psychologists and four facilitators participated in consensus actions. Ten members attended in‐person and three participated through virtual meetings and email correspondence. Each member completed pre‐work activities (philosophical attestation, surveys, case study submissions, pre‐readings) and participated in the virtual education event as a contributing speaker or attendee prior to the formal meeting. See Supporting Information: Appendix 1 for an outline of the full consensus process.

The in‐person consensus meeting was held over two consecutive days and followed with regularly scheduled virtual work group meetings. We developed the consensus statement through implicit methods built on the approaches taken in our previous consensus process (e.g., skilled facilitation, member votes and facilitated discussions) while abiding by rules of consensus developed by the Feeding Matters Task Force (Estrem et al. 2025; Supporting Information: Appendix 2).

### 2.1 PPL Statement

Contributions by authors or consultants with lived experience were not systematically incorporated into the conceptualization or writing of this manuscript.

## 3 Results

Based on the current treatment literature (Table 1) and results of the consensus process, participants arrived at the following shared summary of the psychological approach to treating learned food aversions.

### 3.1 Consensus Statements

1. Exposure to food and the feeding/eating experience is the common core element across psychological interventions for PFD and ARFID. Three additional shared elements are collaborative goal setting, motivation enhancement, and caregiver involvement. These elements support engagement in exposure and the generalization and maintenance of gains.
2. Differences in the selection of specific treatment techniques to incorporate into an intervention are influenced by the level of severity or clinical impairment experienced by the individual, the individual's age and developmental status, and the primary presentation of the feeding/eating difficulty, rather than the ARFID versus PFD diagnostic framework. All four treatment elements are influenced by severity/clinical impairment and age/developmental status, whereas primary presentation only impacts choice of exposure technique and goal setting. See Table 2 for a glossary of terms/definitions and examples of specific treatment techniques.

**TABLE 2:** Psychological strategies for improving feeding and eating: Glossary of terms.

Implementation of exposure *Exposure*: Gradually exposing individuals to situations that trigger anxious or otherwise distressing thoughts and feelings with the aim of helping individuals to remain present in these situations and to reduce the frequency and intensity of their negative emotional response • *In vivo Exposure*: A therapy intervention that involves directly confronting a feared object, situation, or activity in real life. For patients with feeding/eating problems, in vivo exposure involves eating avoided foods, typically in a graduated way, beginning with a level of contact that the patient can tolerate and increasing the expected contact as the patient's distress/anxiety habituate and/or as they become more confident in coping with the discomfort elicited by exposure. In vivo exposure can also involve avoided places and situations, such as eating at a restaurant or with a friend. • *Imaginal exposure*: A therapy intervention where individuals vividly imagine anxiety‐provoking situations or memories in a safe and controlled setting. For example, a patient who avoids social eating situations because of fears of being judged for their “childish” eating would create a “worst‐case” social scenario and vividly imagine it, perhaps with scripting and role‐play with the therapist; A parent of a medically complex child might be encouraged to imagine their child choking during a meal as part of an intervention aimed at reducing the parent's mealtime anxiety and increasing their willingness to tolerate their child's temporary distress during exposures. Re‐scripting exercises might also be incorporated into imaginal exposure. This would involve the patient generating a response to the imaginal scenario a response that reinforces their mastery over the situation or ability to problem‐solve and cope. The child with selective eating might imagine themselves ignoring comments, making a joke, or explaining ARFID to their friends, while the parent could imagine themselves performing the Heimlich maneuver, then resuming the meal or feeding the child their next meal. • *Interoceptive exposure*: A therapy intervention that involves deliberately inducing physical sensations associated with anxiety in a safe and controlled setting. For children with feeding challenges, this may involve inducing feelings of fullness, nausea, or disgust to work on increasing tolerance of these sensations.
*Response prevention* (*escape extinction*): During and after exposure, and in daily life during naturalistic exposures to feared scenarios, the patient resists the urge to leave the situation or engage in behaviors or mental actions that reduce distress without promoting contact with the situation, as these can prevent habituation or learning that the situation is safe and tolerable. In a child who is being fed (as opposed to eating independently), escape extinction could involve non‐removal of the spoon, i.e., continuing to hold the spoon up to the child's mouth until they engage in a target approach (vs. avoidance) behavior such as allowing the spoon to briefly touch their lips. In children who communicate verbally, response prevention may involve agreeing to eliminate or gradually reduce the provision of reassurance, rituals or safety behaviors that reduce anxiety before, during, or after exposure, or to limit avoidance during an exposure (e.g., agree to chew exposure foods a pre‐determined number of times rather than swallowing them whole to avoid the taste/texture). *Systematic desensitization*: Gradually exposing individuals to feared stimuli in increasing levels of difficulty. Unlike other exposure strategies, where the emphasis is on tolerating the distress and mastering the situation regardless of whether distress is reduced by the end of the exposure, a goal of systematic desensitization is for the child to habituate to the exposure scenario such that they no longer find it distressing; this is typically a target before moving on to the next stage. This approach may be used more often in young children who cannot communicate their distress, or willingness to tolerate distress, verbally, or in children of any age who struggle with distress tolerance/self‐regulation when distressed. In the former case, exposure would progress only when behavioral indicators of distress—such as grimacing, gagging, or pushing the food away—are minimized. *Scaffolding*: The process of providing temporary support to help individuals master new skills or behaviors. For example, this could involve using continuous positive reinforcement/distraction during initial exposures to help a child tolerate being in the exposure situation, initiating exposure at a level the psychologist expects the child to be proficient or nearly proficient (e.g., an empty spoon or pea‐sized bite), or providing extra support such as hand‐over‐hand guidance or one‐on‐one attention and coaching during exposure meals. *Fading*: Gradually reducing the degree of prompts and structure (i.e., scaffolding) needed to achieve a behavior. For example, a child may need visual, verbal, and hand over hand prompts to take a bite in early phases of treatment and then these are decreased over time. Reinforcement for target feeding/eating situations is faded as children gain mastery. *Simultaneous presentation*: A preferred and non‐preferred food item are offered at the same time to encourage consumption of the less desirable food item. *Blending*: A less preferred food is blended with a more highly preferred food. Over time, the proportion of preferred food in the blend is gradually reduced until the less preferred food is tolerated on its own. In older children, this is done with the child's understanding and agreement; for example, in a child learning to eat a new pasta shape, gradually increasing the ratio of familiar to new pasta in the bowl over successive meals. *Shaping*: Behaviors are achieved by reinforcing successive approximations of the goal behavior. For example, initial reinforcement for allowing a spoon to be on a table, then for opening mouth for an empty spoon, then for opening with a drop of water on spoon, until arriving at reinforcement for taking an actual bite. *Food rotation*: Serving both preferred and non‐preferred foods as part of a meal, sometimes with a set order the foods need to be consumed in. The child has exposure to the non‐preferred food, followed by periods of easier demand with more highly preferred food and cannot skip exposure to only eat more highly preferred food. *Exposure hierarchy*: A list of planned exposures typically ordered by difficulty. Depending on the child's ability to communicate they may offer input on which tasks they anticipate being easier or harder, and what level of difficulty they are willing to engage with, and the order and pace of exposures may be individually tailored. *Taste tests*: Technique used to design exposure hierarchies and choose exposure tasks. This typically involves presentation of small portions (i.e., bite‐sized pieces) of 2–5 exposure foods from a previously agreed‐upon list, with the child encouraged to take 1–2 bites of each. The goal is to encourage initial exploration and inform the development of a hierarchy. In younger children, behavioral signs of distress may be used to guide the selection of exposure foods or their order on the hierarchy. *5‐step exercise*: A specific exposure technique described in the CBT‐AR manual used during initial taste tests or food exposures in the context of selective eating/sensory sensitivity clinical presentations. During food exposures, patients engage in sensory exploration of the food without making evaluative judgments. The five steps involve describing the appearance, smell, texture (with hands or utensils), taste, and oral texture of a food. In addition to redirecting attention away from hedonic reactions and judgments about preference (which can reinforce the child's expectation of an aversive response), the five steps serve as a graded exposure and are a form of shaping (e.g., guiding the patient through a series of behaviors that increasingly promote oral contact and ingestion of the food) appropriate for patients who communicate verbally. *Food chaining*: The practice of choosing new exposure foods based on sensory similarities to familiar foods. For example, if a child accepts apple sauce, apple slices might be chosen as an exposure food based on their similar taste. Although the expectation is that similarities in some sensory properties might make the exposure more manageable or increase the child's buy‐in, new exposure foods are different enough from preferred foods that exposure still needs to be gradual and progressive.
Motivation enhancement *Psychoeducation*: Orienting the patient and family to the treatment by introducing the explanatory model for the development and maintanence of the symptoms, rationale for interventions (exposure), the expected outcomes of treatment, and the role of the treatment team, family, and patient. The aim is to ensure that the treatment team and family/patient have a shared understanding of the goals and specific tasks involved in treatment. *Motivational interviewing*: A therapy approach that helps individuals resolve their ambivalence about changing a specific behavior by exploring and resolving their uncertainties. It is a patient‐centered, directive method that focuses on enhancing intrinsic motivation to change that is particularly useful when individuals are experiencing high levels of ambivalence, low confidence, low desire or low importance in making a change. *Intrinsic motivation*: The drive to engage in an activity for its inherent satisfaction rather than for external reward or pressure. In the case of feeding/eating problems, this may include physiological goals, such as wanting to gain weight or be stronger, as well as social goals such as wanting to eat with friends and family or wanting to be able to participate in activities that may be limited due to current mealtime behaviors. Hunger can also be considered intrinsic motivation. *Antecedent manipulation*: Changing aspects of the meal or exposure before it begins to help with improving participation and decreasing negative behaviors. *Appetite modification*: Strategies to help increase appetite prior to a meal or exposure to increase likelihood of participation and success. Strategies include medication (e.g., cyproheptadine, mirtazapine, olanzapine), scheduling meals spaced apart with periods where the child does not consume food or drink with calories, or scheduling tube feedings such that the child has a gap to build hunger. *Mealtime hygiene*: Having a predictable structure surrounding meals to help decrease anxiety and make the expectations of meals clear. Examples of mealtime hygiene may include scheduling meals and snacks at specific times, having the child sit for a set amount of time at the meal, family rules surrounding expectations at meals, and being exposed to foods other family members are eating. Eating on a regular schedule also promotes hunger at meals. *Meal enrichment*: Aims to make mealtimes more pleasant and reduce conflict between the child and family by increasing positive interactions (e.g., child‐led conversation, positive emotions and praise) and decreasing negotiating, prompting, and argument during meals. Ultimately, the aim is to reduce negative associations with mealtimes for both child and caregivers, setting the stage for engagement in structured exposures during meals. Techniques include planned ignoring of disruptive behaviors or negotiating, increased positive attention for desirable behavior, and limiting prompting and instruction while using specific, behavioral language in prompts (e.g., “take a bite” versus “please eat something”). *Non‐contingent access to reinforcers*: Used when children are unable to engage in the lowest‐level contact with food (i.e., entering the room for exposure, being present during a meal), this strategy aims to boost positive affect and create positive associations with meals so the child will begin to engage in treatment (usually as a form of scaffolding that is faded out as the child gains mastery). Involves access to a reinforcing toy or activity (such as playing table games during exposure meals) that is not contingent on any engagement in exposure other than remaining present. *Reinforcement*: Strengthening or increasing the likelihood that a specific behavior will occur again in the future. It is a consequence that follows a behavior and makes that behavior more probable *Positive reinforcement of approach*: Providing a positive consequence after a preferred behavior occurs. This may be social attention, praise, or tangible rewards such as access to a preferred toy, or tokens towards a larger reward. How often reinforcement occurs is gradually faded over the course of treatment *Reducing positive reinforcement of avoidance*: Strategies including redirection, planned ignoring of disruptive behavior or attempts to negotiate, and structured prompting/reminders, are aimed at eliminating positive reinforcement—in the form of caregiver attention and engagement—for avoidant mealtime behaviors *Negative reinforcement of approach*: Removing an aversive stimulus after a behavior occurs to increase how often this happens in the future. For example, a child may be allowed to leave the therapy room sooner if they complete the exposure, or have non‐preferred food removed after reaching a certain goal. *Reducing negative reinforcement of avoidance*: Response prevention/escape extinction is designed to reduce avoidant behavior by eliminating negative reinforcement for these behaviors. This is paired with reinforcement of approach behaviors described above.
Enhance learning/generalization of gains *Coping skills*: Healthy strategies and techniques that individuals use to effectively manage distressing and challenging situations. *Distress tolerance*: A set of Dialectical Behavior Therapy (DBT) techniques used to manage intense emotional or physical discomfort without making the situation worse. The goal of these skills is to decrease the intensity of distressing symptoms and cope with overwhelming emotions. *Vocabulary building*: Working with the child and family to learn to identify and label emotions, bodily sensations, and sensory experiences to more accurately describe their experience with food and exposures and how these change over time. For example, providing a list of flavor terms with descriptions to help a child participate in the 5‐Step exercise, or psychoeducation about interceptive sensations like hunger and fullness. *Cognitive interventions* *Cognitive restructuring*: A technique used to teach patients to identify and challenge inaccurate, or unhelpful thoughts and replace them with more neutral and balanced thoughts that promote approach. *Behavioral experiments*: A technique in CBT used to explicitly test an individual's beliefs and predictions in real‐world situations. Externalization: An intervention utilized with the aim of helping individuals and their caregivers to perceive the feeding/eating disorder as something separate, rather than a part of the self. *School coordination*: Working with school personnel on how to implement a plan that is feasible in the school setting and helps the child be able to eat or drink the amount needed to maintain health status.
Parent/caregiver involvement *Parent/Caregiver Coaching*: Working with caregivers on how to implement exposure and coping techniques in situations outside of the therapy setting. This includes practice in a variety of environments, incorporating foods from exposures into regular meals, and working with caregivers on the ways they are responding to children in meals. *Reducing accommodation*: Promote exposure and approach to avoided situations by helping parents identify ways in which they change their own behavior to allow their child to avoid situations that provoke distress. Caregivers may accommodate food avoidance actively (i.e., buying separate food for the child) or by changing their own behavior and family routines (e.g., by choosing only restaurants that serve preferred food, or by opening windows and turning on fans during family meals to prevent the child from smelling food). Like exposure, removal of accommodation is typically gradual and where possible discussed and planned in collaboration with the child. For example, rather than packing their child a separate meal before going to a family party, a caregiver would give their child time to pack the meal or to eat at home after the party. *Parent–child interaction therapy/parent management training*: Evidence‐based, behavioral approaches to young children (under 6 for PCIT) with externalizing behavior problems. These strategies emphasize increasing positive, child‐focused and child‐led interactions between parents and children and the use of positive reinforcement of desired behavior, planned ignoring of undesirable behavior (i.e., removal of reinforcement), and where necessary, the use of consistent, age‐appropriate, and safe disciplinary strategies. In the context of readiness for feeding/eating treatment, these strategies might be recommended when parenting is inconsistent, highly permissive, or potentially unsafe.

Although all participating experts agreed on this shared approach, different terminology used in PFD and ARFID treatment settings can lead to confusion for patients, families, and referring providers. Below is a plain language summary of the overarching treatment approach shared by our members (Sections 1 and 2, Table 2) as well as the mechanisms for integrating specific elements into treatment (Section 3, Table 3).

1. The Common core elements of psychological interventions for PFD and ARFID, defined below, are (a) exposure to food and the feeding/eating experience, (b) collaborative goal setting, (c) motivation enhancement, and (d) caregiver involvement. These elements support engagement in exposure and the generalization and maintenance of gains.
  - Exposure

**TABLE 3:** Integrating treatment‐determining elements in exposure delivery: Illustrative case examples.

CASE 1
Ten‐year‐old patient with early complex medical history necessitating gastrostomy (G) tube since infancy Medically and nutritionally stable Dependent on G‐tube feeding No cognitive or developmental concerns Presents: — Eating a limited variety of foods — With general disinterest in food/low appetite — Psychosocial impairment related to difficulty participating in social activities that involve food and refusal to accept tube feeding during the school day

Variables that Influence Treatment			
	Severity^a^	Developmental level^a^	Presentation^b^
Patient/ family features	*Low medical, nutritional, and psychiatric severity/acuity*	*Low developmental support needs*	*Selective*
	History of medical issues now resolved Weight gain and growth are stable and age appropriate on tube feedings Meeting macro‐ and micro‐nutrient needs on tube feedings No co‐morbid behavioral health conditions	In a typical fourth grade classroom Age typical feeding skills Receives no developmental therapies G‐tube feedings are administered by school nurse during the school day	Eats 6 foods from 2 food groups Will not try new foods Expresses anxiety about trying anything new Gags and expresses disgust when taking a bite of a new food
	*Severe feeding problem*		*Appetite*
	Almost complete dependence on tube feeding Eats from only 2 food groups		Eats ¼ of an age typical portion at most meals Never expresses hunger Rarely enjoys eating Reports feeling full after eating small portions

	Severity	Developmental level	Presentation
Exposure technique	Nutritional stability allows pace of exposure to be set by patient/family. For example, can introduce new foods slowly and gradually while monitoring distress or can focus on increasing flexibility around food rather than targeting ingestion of large/measurable volumes of new foods	Exposure can include a cognitive component (e.g., 5‐step approach, externalizing ARFID, practicing vocabulary to describe the sensory properties of food) Exposure can be to the taste and texture of food and fluid, to the sensation of fullness, and to the experience of social eating situations	Exposure with goal of increasing dietary variety can target disgust, distaste, and aversion to other sensory properties of non‐preferred foods as well as social anxiety about experiencing an aversive response when trying a new food Exposure with goal of increasing the volume of food consumed by eating past signs of fullness can target visceral sensations, feelings of fullness, and emotional stress around mealtimes
Goals	Because there is no immediate danger to safety, goals can be strongly influenced by the patient and family, rather than heavily influenced by the clinician Goals can be tailored to the patient/family's reason for seeking treatment, rather than directed by the clinician	Although the primary treatment goal of the clinician may be tube weaning, patient and family may opt to set goals around developmentally typical feeding. For example, with the support of G tube feeding, the patient with low support needs may want to target social integration (i.e., learning to eat with peers) rather than consumption of age typical portions or oral consumption of high calorie beverages to facilitate tube weaning.	Goals may be to teach child and/or caregivers strategies to introduce and incorporate new foods into meals independently to increase dietary variety Goals may be to gradually and systematically increase the amount of food eaten while simultaneously (coordinating care with dietitian) decreasing supplemental tube feeding to establish intake of an adequate volume of a nutritionally‐complete diet
Motivation	Because weight and nutrition are stable, the patient's engagement and buy‐in can be considered. If patient and family are not interested in change, it might be best to defer treatment until they are ready for change. Time may be spent in pre‐treatment evaluating this readiness for change.	Because of the patient's chronological and cognitive age, internal motivation may be present and can be used to enhance engagement. Most school‐aged children require external motivation. Techniques for a patient this age can include tokens toward earning a larger reward or access to certain preferred activities contingent on participation.	*Motivation enhancement strategies and the importance of child vs. parental motivation is not dependent on the primary presentation of the feeding/eating problem.
Parent involvement	Because of medical and nutritional stability, parent involvement can be flexible and at the discretion of the patient and the clinician. However, most school‐aged children require family support and participation to generalize and maintain gains.	Parent involvement can be in line with what is typical for chronological age (e.g., reminding to do therapy “homework”, helping to run exposure at home, implementing contingencies)	*The degree and nature of caregiver involvement is not determined by the primary presentation of the feeding/eating problem.

CASE 2
16‐year‐old patient with autism and moderate intellectual disability, severe expressive and receptive language deficits. No significant medical history Acute onset of food refusal associated with rapid weight loss. Food refusal was precipitated by choking on a tortilla chip Before the choking incident, ate a relatively narrow but nutritionally complete and age‐appropriate diet with no reported eating‐related impairment Presents restricting all solid foods requiring chewing Dependent on oral nutritional supplements and high calorie meal replacements but still losing weight due to difficulty consuming enough calories from a fully liquid diet

Variables that influence treatment			
	Severity/acuity	Developmental level	Presentation
Patient/family features	*High medical, nutritional, and psychiatric severity/acuity, severe feeding/eating problem*	*High support needs*	*Fear*
	Losing or unable to maintain weight Inadequate oral intake and weight loss put patient at risk for malnutrition The eating problem had an acute onset and involved a dramatic change in eating behavior compared to the patient's baseline	Severe expressive and receptive language deficits Moderate intellectual disability	The patient experienced a frightening incident involving eating/the GI tract (in this case, choking on a chip), and rapidly acquired an intense fear of the experience itself and of associated interoceptive sensations in the GI tract (in this case, throat).
			*Selectivity*
			The patient was described as a “picky eater” before the incident but was able to eat an adequate volume and variety of foods for growth and nutrition and did not experience psychosocial impairment related to their eating. Therefore, selective eating is not judged to contribute to ARFID symptoms in general or to the current illness.

	Severity	Developmental level	Presentation
Exposure technique	Improving nutritional status takes priority over directly intervening on the problematic eating behavior (refusing solids). Typically this is done in the least invasive way tolerated (e.g., beginning with oral before moving to enteral supplemental nutrition). Any exposure that does take place early in treatment is in the service of introducing and/or increasing nutritionally complete drinks. If the patient cannot tolerate the pace of exposure necessary to restore adequate oral nutrition, enteral nutrition might be needed to allow for more gradual exposure to eating by mouth while still stabilizing the patient	Exposure for patients with high support needs for language and cognition (or young chronological age) involves systematic exposure beginning with low demands. The difficulty of exposure is increased after the patient behaviorally demonstrates that they have habituated to/learned to trust the current exposure situation (e.g., after 15 successful bites with no avoidant behavior or visible distress).	The feared consequence maintaining eating restrictions is (inferred to be) choking. In this case, exposure would focus on increasing texture/difficulty of chewing.
Goals	The initial goal is to stabilize the patient's weight and nutritional status by increasing caloric intake to a level needed to maintain or gain weight and achieve adequate nutrition.	Goal setting considers the patients’ baseline abilities and support needs. In this case, the patient had eating skills (i.e., chewing and swallowing age‐appropriate textures) that were consistent with their chronological age, but their skills and/or interest in, for example, trying new foods, eating with peers or learning to prepare food independently may not be consistent with chronological age, and so there may not be goals in these areas.	After weight and nutrition are stabilized, returning to this patient's pre‐morbid eating baseline is likely to be a realistic end‐of‐treatment goal, given the acuity of the feeding/eating problem and the relatively short illness duration.
Motivation	Given the severity of the eating/feeding problem and its impact on weight and nutrition, some degree of intervention and change is necessary regardless of the patients’ motivation. If the patient cannot be motivated to participate in exposure therapy, they are likely to require enteral feeding for acute stabilization. This is true regardless of the patients’ chronological age and support needs. Motivation enhancement strategies such as motivational interviewing or “creating an intense scene” (FBT) may be used with caregivers who will be supporting or delivering the treatment.	For youth with high support needs (or young chronological age) like this patient, it may not be possible to assess the degree of internal motivation for change, and motivation enhancement strategies are likely to be reinforcement‐based and relatively immediate, with rewards given at short intervals or with every instance of the desired behavior. Typical rewards include access to a favorite toy or activity after each interval or instance of the desired behavior. In some cases, patients have continuous access to reinforcers if they continue to participate in the exposure (e.g., having their tablet or a favorite toy on the table during exposure meals).	*Motivation enhancement strategies and the importance of child vs. parental motivation is not dependent on the primary presentation of the feeding/eating problem.
Parent involvement	When the impact of food‐avoidant behavior is severe, parental involvement is usually necessary to achieve the necessary rapid change by removing accommodation and delivering exposure consistently and frequently at home. If parents are unable to do this, outpatient treatment is not appropriate.	For patients with higher support needs (and young chronological age), therapy is often caregiver directed and employs the “parent as therapist” model. Caregivers are taught to structure meals and conduct exposures at home to promote generalization.	*The degree and nature of caregiver involvement is not determined by the primary presentation of the feeding/eating problem.

^a^ “Severity/acuity” and “developmental level” are on a continuum (e.g., “low severity to high severity” or “few support needs to many support needs”).

^b^ Feeding presentations are not mutually exclusive.

The explanatory model supporting the use of exposure‐based PFD/ARFID treatment is that clinically impairing food avoidance is acquired and maintained by associative/classical and operant learning. In some cases, associative learning occurs when a medical condition or mismatch between the situation and the child's feeding/eating skills causes pain, discomfort (i.e., uncomfortable fullness, breathlessness), distress, or frustration when feeding/eating. Food avoidance persists even when the original aversive experience is no longer occurring because of a learned association between food and feeding/eating and distress or discomfort. Aversive responses to food may also be acquired via one‐time experiences (e.g., one episode of accidental choking) or by modeling or vicarious learning (e.g., seeing another child vomit or have an allergic reaction). In other cases, temperamental risk factors that influence how the individual interacts with and perceives their environment (e.g., heightened sensory sensitivity or satiety awareness, anxiety‐proneness, the tendency to eat less when emotionally distressed) may be enough to produce initial aversive reactions that are strengthened and reinforced over time by associative processes. For example, a child with enhanced visceral satiety awareness having multiple experiences of being pushed to eat past fullness, resulting in physical discomfort and emotional distress, learns that eating age‐typical portions is aversive. Operant learning involves engaging in a behavior because it has been positively or negatively reinforced. For example, avoiding food may be positively reinforced by increased caregiver attention or negatively reinforced by the individual circumventing the expected aversive emotional or physical response to feeding/eating.

The psychologist's goal in treating feeding/eating avoidance is to address maladaptive associative and operant learning by helping the individual tolerate increasing levels of contact with avoided food and feeding/eating situations (i.e., exposure). The desired result of exposure is consumption of an increased variety and/or volume of food and fluid and reduced negative affect or refusal to eat during meals. Table 2 defines specific exposure techniques. Exposures are designed to elicit the distressing aversive emotional/physical response that maintains problematic avoidance of food. By experiencing a feared situation and the accompanying aversive emotional response in a controlled setting, children learn that the situation is safe (e.g., the feared outcome involved in the etiology of the aversive learning will not occur or can be tolerated) and that they are able to tolerate and cope with the aversive emotional response.

We note that the learning framework described above is not a hypothesis for the full etiology of each ARFID presentation. Although classical and operant conditioning mechanisms are the primary targets of exposure‐based therapy, individual neurobiological and psychophysiological differences likely explain a great deal of risk for and maintenance of each ARFID presentation. Non‐environmental etiological and mechanistic factors include cognitive rigidity and sensory sensitivity (selectivity), homeostatic regulation and reward processing (appetite), and individual differences in propensity towards developing and maintaining conditioned fear associations along with risk factors related to aversive experiences themselves (e.g., food allergy, gastrointestinal disorder, visceral hypersensitivity; fear) (Thomas et al. 2017; Thomas et al. 2025).

b Goal setting

Evidence‐based psychological treatments are goal‐directed and time‐limited (Chambless and Hollon 1998). As such, goals for feeding/eating aversion interventions are discussed and agreed upon at the start of treatment by the treatment team, patient, and family and are continually updated as progress is monitored and assessed. There is a shared expectation that treatment will produce meaningful change, which may or may not be complete elimination of the feeding/eating problem. Regardless, the goal is to teach the patient and family how to establish new routines and expectations around food and feeding/eating and to identify techniques that work to improve feeding/eating and drinking in the home and community environments using skills learned in treatment, which are meant to continue after treatment ends.

c Motivation

Because exposure requires engagement from the patient to be effective, psychologists use a range of techniques to enhance patients' and caregivers' motivation to participate in treatment. These techniques may be aimed broadly at boosting their intrinsic motivation to initiate treatment and to change their feeding/eating behavior or specifically at enhancing the patient's willingness to engage in each exposure, or both.

d Caregiver involvement

Although the child is the patient, treating feeding/eating problems in children typically involves the caregivers. Involving caregivers is crucial for learning to generalize beyond the treatment setting and continue at home. It also provides caregivers with the tools necessary to encourage their child to confront difficult situations with the long‐term goal of achieving feeding/eating improvements.

Caregiver involvement may include education about the nature of the feeding/eating problem, instruction on how to support/structure feeding and eating at home, direction on how to support at‐home exposure practice, and modification of caregiver emotional and behavioral responses to feeding and eating behavior.

2 Factors that influence how these common elements are incorporated into treatment are: (a) the severity/acuity of symptoms, (b) the patient's age and developmental status, and (c) the presentation of feeding/eating problems. These three influential factors, defined below, are assessed prior to beginning psychological interventions typically as part of a multidisciplinary evaluation.
  - Severity/acuity

Severity and acuity of the feeding/eating problem can manifest in multiple domains. These include medical/nutritional severity (e.g., risk to the patient's life, health, or ability to achieve their personal height/weight potential), psychiatric severity (e.g., acute suicidality, cognitive or affective symptoms that interfere with participation in treatment, level of distress associated with the aversive responses to food), the severity of the feeding/eating problem itself (e.g., significant worsening from the child's baseline eating/feeding to current, significant difference from their same‐age peers), and the timing of onset (e.g., longstanding feeding/eating difficulties, gradual deterioration in feeding/eating over time, sudden onset of feeding/eating problems).

b Age/developmental status

In addition to chronological age, a child's developmental status is considered when designing feeding/eating intervention. During the initial assessment process, patients' feeding/eating skills, capacity for self‐regulation, meta‐cognitive status, communication capacity, and comprehension abilities are formally and informally evaluated.

c Presentation of the feeding/eating problems

As described in the Introduction, feeding/eating problems documented in the literature can be characterized broadly as (1) selective eating, food neophobia, and disgust or distaste responses, (2) poor appetite, lack of interest in food or eating, and preference for other activities over eating, and/or (3) fear of food or eating.

3 Treatment‐influencing factors (severity, age/developmental status, and the feeding/eating presentation) interact to impact how the core elements of intervention are implemented (exposure, goal setting, motivation enhancement, and caregiver involvement). See Table 3 for two illustrative case examples.
  - Exposure

The techniques used to deliver exposure and the pace of the exposure vary by severity of the feeding/eating problem, the patient's developmental status, and the presentation of the feeding/eating problem. For example, in patients with lower medical or psychiatric severity the pace and intensity of exposure and the specific situations and emotions targeted can be determined by the patient's stated goals and their willingness to experience distress; exposure does not have to be driven by medical or nutritional concerns. However, when symptoms are severe and acute, exposures targeting the emotions and behaviors that are maintaining dangerous symptoms are prioritized and may need to be intensified or enhanced.

The pace and techniques used to deliver exposure vary across the continuum of developmental status. In young children and those with delayed/impaired metacognitive and communication abilities, exposure is delivered as a primarily behavioral intervention, with the level of contact with the feared stimulus gradually increased as the child's observable aversive responses and avoidant behaviors decline. Older children who communicate verbally are encouraged to describe their emotional response, and their verbal report of their own distress is used instead of, or along with, observed behavior to guide decisions around increasing the intensity of exposure. Developmentally appropriate cognitive interventions complement exposure and reinforce new learning. These cognitive interventions can include encouraging exploration and curiosity about unfamiliar food‐ and feeding/eating‐related situations including the development of new vocabulary to describe once‐avoided tastes, textures, and interoceptive sensations (e.g., Thomas and Eddy 2018; Zucker et al. 2019), and engaging in post‐exposure processing to highlight the discrepancy between what the child feared would happen and what they actually experienced (e.g., Dumont et al. 2019; Lane‐Loney et al. 2022; Thomas and Eddy 2018).

Finally, the nature of the aversive emotional response targeted by exposure depends on the presentation of the feeding/eating problem. For example, if poor appetite or limited interest in food is the primary presenting problem, tolerance of visceral sensations/feelings of fullness may be the primary target of the exposure intervention. If food selectivity is of greatest concern, exposure may target tolerance of disgust and/or habituation of distaste towards the sensory properties of food. Exposure for fear of food generally involves the expectation that the feared outcome is unlikely to occur or can be tolerated if it does occur (e.g., that typical eating situations are “safe”).

b Goal setting

The specific behavioral and emotional change goals being set depend on severity, age, and the presentation of the feeding/eating problem. More specifically, when impairment is severe, goals are determined by the patient's safety needs. When there is no immediate danger to safety, goal setting involves more patient and/or family input and is more heavily dependent on family and/or patient preferences.

The child's age and developmental abilities can influence the scope of goals. For example, children at an older developmental age can play a more prominent role in setting and establishing goals, where children who are younger or have more severe cognitive delays may have limited explicit participation in long term goal setting.

Finally, the specific presentation(s) of the feeding/eating problem are delineated by the patient and the clinician during the early phases of treatment and are incorporated into treatment goals. For example, if the primary presentation involves selectivity, goals would focus on increasing food variety and tolerating or managing disgust; if the primary presentation involves poor appetite, goals will focus on increasing the amount of food and fluid consumed and tolerating or managing fullness; and if the primary presentation involves fear, goals might focus on (re)establishing acceptance of foods and/or eating situations avoided because of anxiety.

c Motivation to participate in exposure

The specific strategies for enhancing patient motivation depend on symptom severity and age/developmental status but do not differ by the primary presentation of the feeding/eating concern (i.e., similar motivation enhancement strategies are used whether addressing selectivity/limited variety, poor appetite/limited volume, or fear of aversive consequences/food avoidance). Psychologists may enhance motivation by working with the child and their family to create incentives for engaging in exposure and/or by using open‐ended questioning to help the patient and family articulate the reasons they desire change.

The severity and acuity of symptoms influence the use of motivation enhancement strategies and type of strategies used. For example, when symptoms are not medically or psychiatrically dangerous, patients and families who are not intrinsically motivated to engage in treatment might be encouraged to defer treatment until they are more ready for change (i.e., limited use of motivation enhancement strategies by the clinician). However, if symptoms are sufficiently severe, incentives must be incorporated into treatment, for example, offering continuous reinforcement for participating in exposures (e.g., having access to a tablet or preferred toy during exposure meals) or making privileges contingent on participating in the intervention (e.g., attending extracurricular activities only if eating a minimally adequate amount of food).

Age and degree of cognitive development are important considerations in determining the schedule and nature of reinforcement. For example, at the earliest stages of development, rewards are tangible and immediate (e.g., clapping, cheering, and providing access to a toy after each bite of food eaten), while more distal concrete rewards (e.g., a token economy, a future trip) or valued aspirations (e.g., getting stronger for sports) are more effective motivators for older children.

d Caregiver involvement

Symptom acuity and developmental status influence the degree of caregiver/family involvement needed for treatment to be effective. Specifically, a high degree of caregiver involvement and directiveness is warranted for patients who present with higher severity of medical or psychiatric impairment, particularly in the context of low motivation to engage in treatment. In cases like these, caregivers rather than the patient themselves may be the main target of the intervention. For patients with lower medical or psychiatric severity, the degree to which caregivers are involved may be flexible and dependent on family functioning and availability, the patient's motivation to address the feeding/eating problem, and overall degree of independence in other areas of life.

Considering age, developmentally younger or delayed patients require structure from caregivers to initiate and execute exposures, and caregivers must implement contingencies to enhance/maintain motivation. Individuals with lower support needs may not require as much caregiver support to participate in exposure‐based intervention.

The case examples in Table 3 (above) illustrate the complexity of integrating these three considerations into treatment decision‐making, as each exists on a continuum and the three dimensions are orthogonal. Further, there is heterogeneity within dimensions. Severity encompasses the eating/feeding problem itself as well as its functional impact. Case 1 presents with a severe feeding problem, in that they are nearly fully dependent on enteral feeding; however, these problems are chronic and they present as nutritionally stable (not severe). This stability allows the clinician to carefully consider the patient and family's readiness and motivation for treatment. The severity of Case 2's presentation (acute onset of eating restrictions with adverse impact on weight and nutrition) necessitates a rapid pace of change that requires significant motivation enhancement for participation. Goals will necessarily focus on weight and nutritional rehabilitation. Regarding developmental status, Case 2 presented for treatment with a history of age‐typical eating skills despite limited communication abilities and intellectual disability, whereas Case 1, while typically developing in other areas, had a limited history of eating by mouth and may present with some eating skill or knowledge deficits. Both children were described as selective or “picky” eaters, but in Case 2 this was determined not to be a clinically significant contributor to impairment.

## 4 Discussion

The consensus from the 2024 meeting was that the primary evidence‐based approach to psychological treatment of food aversion and avoidance is exposure and presumes that food avoidance is acquired through associative/classical and operant conditioning (Estrem et al. 2025). Evidence‐based psychological interventions target learned emotional responses and behaviors in patients diagnosed with PFD or ARFID. Psychological interventions for PFD and ARFID share additional treatment elements including goal setting, establishing motivation to participate in intervention, and caregiver involvement. The perceived practice differences among experts working with patients with pediatric feeding and eating problems are merely differences in terminology and the specific combinations of treatment techniques incorporated into practice. The choice of techniques used to deliver each treatment element, and their combination into an individualized treatment plan, depends on the severity and acuity of the feeding/eating problem and its consequences, the age or developmental status of the patient, and the presenting feeding/eating problem.

Members highlighted several other important aspects of treating feeding/eating problems in addition to the core psychological treatment of exposure and outside of the formal consensus process. Whereas evidence from recent systematic reviews (e.g., Willmott et al. 2024) and universal agreement among consensus participants supports the importance of exposure as a core intervention and goal setting, motivation enhancement, and caregiver support as crucial elements in its delivery, there was less agreement on best practices and less available empirical evidence for the following considerations. Nevertheless, we agree that they are important and warrant further study and systemization. These include conducting multidisciplinary assessment prior to treatment, focusing on a patient and family's readiness for treatment, and assessing and addressing systemic factors that influence access to and course of treatment.

Prior to treatment, multidisciplinary assessment is recommended to (1) determine if other interventions (e.g., medical, feeding/eating skill‐based) are necessary to resolve or appropriately manage contributors (e.g., medical conditions or oral/motor factors that cause pain, discomfort, or frustration) and prevent further worsening of the food avoidance or aversion (2) assess treatment determining factors (severity/acuity of medical, nutritional, and psychiatric symptoms, developmental status, and the presentation of the feeding concern), and (3) determine the appropriate level of care (Eddy et al. 2019; Estrem et al. 2025). In many specialized programs treating feeding/eating problems, multidisciplinary assessment is a standard and universal element of treatment; however, this may not always be readily available in the community. When multidisciplinary assessment is not available, patients with feeding/eating problems should be evaluated by the referring provider (e.g., primary care physician or pediatric specialist) to confirm that medical and skills‐based contributors to the eating/feeding problem are absent or appropriately managed (e.g., Eddy et al. 2019).

Once referral for psychological intervention is made, clinicians and families engage in preparation for treatment. This collaborative process is meant to ensure that a family is ready for treatment and able to participate in intervention. Two potential ways a family may not be ready for intervention are difficulty tolerating distress and the use of parenting strategies that are incompatible with the exposure‐based approach that is common to feeding/eating treatment.

Effective exposure‐based intervention causes some level of distress or discomfort in that core to the therapy is overcoming fear while learning to approach rather than avoid distressing situations. However, distress that becomes overwhelming and unmanageable is counterproductive and has the potential to reinforce fear and avoidance. Therefore, part of pre‐treatment work is assessing the patient and/or caregivers' ability to tolerate and cope with distress and ensuring that they are aware of the role of temporary distress in exposure therapy. Building and practicing distress tolerance and coping skills prior to and throughout feeding/eating intervention can enhance the effects of exposure‐based treatment by helping the patient and family to engage in the intervention more successfully.

As needed, pre‐treatment work can also focus on modifying parenting strategies to better align with those that best support feeding/eating interventions. This may require a trauma‐informed approach, specifically considering parenting styles that have developed in response to a child's developmental differences, medical complexity, or feeding and eating difficulties. As well, parenting styles that are not compatible with consistent and safe application of exposure therapy (i.e., parenting that is overly harsh or permissive in areas other than feeding and eating) can be addressed using evidence‐based interventions like Parent Management Training or Parent/Child Interaction Therapy (e.g., Helander et al. 2024; Thomas et al. 2017). As with building distress tolerance, focus on parenting strategies outside of mealtimes can enhance a patient and family's response to exposure‐based intervention for feeding/eating.

Finally, consensus members emphasized the importance of recognizing a family's relationship to larger systems, specifically identifying systemic factors that may present barriers to accessing evidence‐based treatment or factors that facilitate access. Consensus members identified culture and spiritual practices and socioeconomic status as particularly important systems to consider in collaborative goal setting throughout treatment. Cultural and spiritual preferences can influence food choices, mealtime routines, feeding/eating style, and openness to medical intervention, among many other factors that directly inform the family's goals for treatment. Additionally, stigma and cultural beliefs about psychological care may affect a family's willingness to pursue treatment. Socioeconomic disparities can impact a patient's access to food or to specialized formulas, caregiver availability to participate in treatment and/or support feeding/eating at home, and insurance benefits and access to treatment itself. Geographic location can affect access to evidence‐based behavioral health care, with the availability of a tertiary feeding/eating disorder‐focused program determining whether a given child is treated by a team specifically trained to treat PFD and/or ARFID or by less specialized providers. Each of these factors can lead to decreased utilization of psychological intervention for feeding and eating concerns and, at times, variable adherence to recommendations made by interventionists.

Although the consensus participants concluded that psychological treatment for feeding/eating problems is based on a shared explanatory model and intervention approach, regardless of whether the feeding problems are conceptualized as ARFID or PFD, we also recognize that most programs treating feeding/eating problems are designed around the intervention techniques most appropriate for younger patients and patients with high support needs such as individuals with autism (PFD programs) or older and more psychiatrically complex patients (eating disorder programs). Indeed, most consensus participants represented programs that present themselves as specializing in either PFD or eating disorders (including ARFID). Despite our shared understanding of ARFID/PFD psychopathology, the fact remains that not all patients presenting with feeding/eating problems are ideal candidates for treatment in all feeding/eating programs, as different programs specialize in different aspects of intervention. Thus, when choosing where to refer a child with feeding and eating challenges, it is beneficial to learn about the ages, developmental levels, and presenting problems typically treated by the program more than whether the program specifies working with children with ARFID or PFD.

## 5 Conclusion

The aim of the consensus was to identify similarities and differences in clinical practices between ARFID and PFD‐focused treatment approaches. During the consensus process, we identified a shared explanatory model and overarching treatment approach between the two fields. It is important to note that this consensus was not the result of a systematic review to evaluate the evidence base. Rather, we developed the consensus statement through a discussion of empirical research supported by systematic reviews (e.g., Willmott et al. 2024), learning theory, and current best practices as our consensus participants interpret them. In coming years, we expect to see the publication of clinical trials and mechanistic psychopathology studies designed and powered to probe hypothesized maintenance mechanisms of feeding/eating aversions. In response, some of the recommendations in this statement may be strengthened or amended.

We identified a need for a common language to describe our shared understanding of the etiology and treatment of pediatric feeding/eating problems. We hope that improved communication will allow clinicians and researchers to better serve children and families with these overlapping conditions. This work represents a step towards a more unified approach to the care of these patients that will lead to the development of tailored evidence‐based treatments and referral pathways that consider developmental status, clinical severity, and behavioral presentation. Finally, this enhanced communication may promote collaboration with other domains of psychology (e.g., developmental psychology, pediatric psychology, infant mental health) during exposure‐based interventions to further enrich and tailor the psychological services provided to patients with eating/feeding disorders and their families.

## Author Contributions

All authors were involved in conceptualization, writing review and editing, and writing original draft. Jaclyn L. Pederson and Cuyler Romeo were involved in funding acquisition and developing the methodology. Colleen T. Lukens, Jacklyn L. Pedersen, Cuyler Romeo, and Hana F. Zickgraf were involved in project administration.

## Funding

Funding was provided by Feeding Matters.

## Conflicts of Interest

Drs. Thomas and Eddy receive royalties from Cambridge University Press and Routledge for the sale of their books. Dr. Thomas also receives royalties from Oxford University Press for the sale of her books. Drs. Thomas and Eddy receive consulting fees from Equip Health. D. Zucker receives royalties from Cambridge University Press for work related to this topic. Dr. Menzel owns stock in Equip Health. Ms. Pederson is the CEO of Feeding Matters, and Ms. Romeo is Feeding Matters' Director of Strategic Initiatives. The other authors declare no conflicts of interest.

## Supporting information


**Figure S1:** Rules of consensus.

**Data S1:** 2024 PFD‐ARFID psychology consensus.

## Data Availability

Data sharing not applicable to this article as no datasets were generated or analysed during the current study.
