# Supplementary material for: Psychological Treatment for Pediatric Feeding Disorder (PFD) and Avoidant/Restrictive Food Intake Disorder (ARFID)
Source: Int J Eat Disord. 2026 May 24;59(9):1929–44. doi: 10.1002/eat.70101 (PMC13536239; doi:10.1002/eat.70101)
Supplement: Supplementary file 1 [file EAT-59-1929-s001.odt]

2024 PFD-ARFID Psychology Consensus
Methods	
Pre-Summit
Feeding Matters Research Initiatives Task Force (RITF) meeting planning
Topics of interest
The 2023 Feeding Matters consensus meeting notes were reviewed for potential topics for the next consensus. RITF members engaged in extended discussions on “future directions” for research and the field. 
Member nomination and selection
The inclusion criteria for participation were authored. 
Consensus members were invited to participate.
Members consented to abide by rules of consensus (Figure 1) and to uphold Feeding Matters philosophy (Figure 2).
Pre-work
Each member was required to submit a bio, case study and article for the consensus member pre-read packet. 
Members also completed an ARFID-PFD Summit Pre-work survey
A compiled document including all member’s pre-work submissions was distributed to each consensus member. 
Additionally, members were asked to review the Feeding Matters 2023 International Pediatric Feeding Disorder Conference presentation on the 2024 PFD-ARFID consensus publication.
Survey results were reviewed by the identified meeting facilitators and used for meeting planning. 

ARFID-PFD Summit Day 1 
Introduction and establishment of meeting structure
Welcome and introductions
Overview of consensus and philosophy
Facilitators verified agreement on rules of consensus (Figure 1) as well as awareness and alignment on the Feeding Matters philosophy (https://www.feedingmatters.org/collaboration-and-philosophy/).
Meet the members
Consensus members gave introductions and shared their experience related to PFD and ARFID.
Overview of methodology
Members were briefed on the consensus process, to include round robin discussion, voting, sharing of counter arguments, final vote, once consensus is reached progress to next point of discussion.
Establishment of team norms
Members were given the opportunity to request additional norms or rules be added to the meeting. No additional norms or rules were requested. 
Determining consensus aim: past, present and future needs
Past consensus review
Facilitators gauged agreement on the 2023 PFD-ARFID 10 consensus statements through dot-voting followed by discussion.
Lessons from the community
Members recorded their top three takeaways from the Feeding and Eating Psychology Summit education event; open discussion followed.
Reformation of the past
Members took a deeper look into the community’s impression of psychological intervention and compared this impression to current treatment. The community’s perception of psychological treatment evolution was discussed. 
Idea generation
Each member was asked to submit their top idea for what would evoke the most change in the field to help inform the next step in consensus.  
ideas were recorded and sorted by level of agreement.
Aim selection – Members identified the need to describe interventions used in PFD and ARFID with a future goal of creating a shared language across the two diagnoses.
Intervention- mapping interventions across diagnoses
Intervention identification by diagnosis 
The consensus team broke into two groups with experts assigned by primary area of expertise/population: those whose research and clinical work focuses on patients with a PFD diagnosis and those focused on patients with an ARFID diagnosis. 
Each group created a list of core psychological interventions used to treat feeding/eating problems in their context. 
Intervention overlap
Lists were exchanged and reviewed by the other population group. 
Through the discussion that followed, we agreed that each group’s list of interventions also applied to the other group’s population (i.e., PFD, ARFID), sometimes with different terminology used to describe the intervention
The full consensus team reconvened and identified shared terms and definitions for each intervention. The list generated through this discussion was the basis for the glossary (Table 2).
Intervention selection
The facilitators guided a discussion on methodology of intervention selection.
The consensus team then discussed the impact of medical and feeding skill factors on intervention selection and application.
Intervention- factors that influence selection
Intervention determination
Members then create a list of patient factors that are considered or formally assessed when matching intervention to patient or family needs. The three primary factors identified by the consensus group were age/developmental status, severity of feeding/eating problem and impact, and the presentation of the feeding/eating problem (Table 3).
Facilitators lead a discussion of perceived effectiveness across both diagnoses.
Medical and feeding skill domains were revisited as key influencers in PFD with ongoing need to rule out deficits in ARFID.
Wrap up
Findings were summarized and members were prepared for the next day’s agenda. 

ARFID-PFD Summit Day 2  
Regroup
Members were welcomed back. 
Each member shared one word that described how they felt entering the meeting.
The facilitators revisited the rules of consensus and restated the goals of the meeting.
Goal 1: publication of a manuscript describing interventions used to treat psychosocial domain in PFD and ARFID
Goal 2: field advancement
The facilitators aided in the identification of the intended audience through open discussion.
Target 1: primary care providers- front line providers for PFD and ARFID
Target 2: providers at large  
Intervention Continued
The facilitators oriented the consensus team to the International Classification of Functioning Disability and Health the Common Report Form to assess intervention list for comprehensiveness. Members broke into groups by diagnosis and used tools to work through a typical assessment process.
Members discussed if listed interventions comprehensively addressed all points of the diagnostic criteria.
Family interventions were discussed in more depth. Members were asked if interventions included family education needs and integration into family routines.
Possible publication topics were revisited, and a central aim was solidified. Members agreed to focus on the identification of shared and unique interventions by diagnosis with a long-term goal of creating a shared language to guide research and to help determine best practices.
A vote was taken, and this aim was agreed upon unanimously. 
The facilitators gathered research aspirations from each member present if no barriers to research or programming existed (20-year outlook) (Figure 6).
The meeting closed with each team member sharing a “one- word” description of their experience. 
Publication
The facilitators asked for volunteers to serve as lead authors. Hana Zickgraf and Colleen Lukens volunteered and were accepted as leads by the consensus group.
Weekly lead author meetings and monthly consensus team meetings were established.  
The first meeting was scheduled and an invitation for a Zoom meeting was sent for October 7th, 2024.
Wrap up
The list of future needs was revisited with discussion regarding which was most important considering the healthcare climate and the community’s feedback and needs.
The facilitators solicited feedback from consensus members through round robin sharing.
Thanks were expressed for all team members. A group photo was taken. 
ARFID-PFD Post-Summit
Completed
The Research Initiatives Task Force was debriefed.
Facilitators reviewed pooled summit notes, dot voting, consensus points, and discussion points. 
The facilitators and lead authors identified the publisher, article type and set a general timeline.
Lead author meetings were scheduled and completed weekly to monthly from September 2024 through December 2025. 
Consensus team meetings were scheduled and completed monthly to quarterly from October 2024 through December 2025. 
The lead authors authored manuscript with feedback solicitation from consensus team with each iteration. The final draft was prepared for submission.
