# Supplementary material for: Psychological Treatment for Pediatric Feeding Disorder (PFD) and Avoidant/Restrictive Food Intake Disorder (ARFID)
Source: Int J Eat Disord. 2026 May 24;59(9):1929–44. doi: 10.1002/eat.70101 (PMC13536239; doi:10.1002/eat.70101)
Supplement: Supplementary file 2 [file EAT-59-1929-s002.docx]

Figure 1 - Rules of Consensus
